# Supplementary material for: Predictions and experimental tests of a new biophysical model of the mammalian respiratory oscillator
Source: eLife. 2022 Jul 7;11:e74762. doi: 10.7554/eLife.74762 (PMC9262387; doi:10.7554/eLife.74762)
Supplement: Transparent reporting form [file elife-74762-transrepform1.pdf]

Westbrook Centre
Milton Road
Cambridge CB4 1YG, UK

P 01223 855340
W elifesciences.org
T @elife

eLife's transparent reporting form

We encourage authors to provide detailed information *within their submission* to facilitate the interpretation and replication of experiments. Authors can upload supporting documentation to indicate the use of appropriate reporting guidelines for health-related research (see EQUATOR Network), life science research (see the BioSharing Information Resource), or the ARRIVE guidelines for reporting work involving animal research. Where applicable, authors should refer to any relevant reporting standards documents in this form.

If you have any questions, please consult our Journal Policies and/or contact us: editorial@elifesciences.org.

Sample-size estimation

- You should state whether an appropriate sample size was computed when the study was being designed
- You should state the statistical method of sample size computation and any required assumptions
- If no explicit power analysis was used, you should describe how you decided what sample (replicate) size (number) to use

Please outline where this information can be found within the submission (e.g., sections or figure legends), or explain why this information doesn't apply to your submission:

Sample size for each experiment was chosen based on existing literature (Tan W, *et al*., *Nat Neurosci* 2008, Sherman D, *et al*., *Nat Neurosci* 2015, Koizumi H, *et al*., *eNeuro* 2016, Malherios-Lima MR, *et al*., *Pflugers Arch*, 2018). Given the large and consistent effects we observed, our sample sizes are appropriate and not underpowered.

Information on the number of replicates for each experiment is reported in the Results section.

Replicates

- You should report how often each experiment was performed
- You should include a definition of biological versus technical replication
- The data obtained should be provided and sufficient information should be provided to indicate the number of independent biological and/or technical replicates
- If you encountered any outliers, you should describe how these were handled
- Criteria for exclusion/inclusion of data should be clearly stated
- High-throughput sequence data should be uploaded before submission, with a private link for reviewers provided (these are available from both GEO and ArrayExpress)

Please outline where this information can be found within the submission (e.g., sections or figure legends), or explain why this information doesn't apply to your submission:

We reported how often each experiment was performed in the Results section. We provided all of the experimental data in the uploaded Source data file.

Data were not excluded as outliers unless warranted by technical issues.

Statistical reporting

- Statistical analysis methods should be described and justified
- Raw data should be presented in figures whenever informative to do so (typically when N per group is less than 10)
- For each experiment, you should identify the statistical tests used, exact values of N, definitions of center, methods of multiple test correction, and dispersion and precision measures (e.g., mean, median, SD, SEM, confidence intervals; and, for the major substantive results, a measure of effect size (e.g., Pearson's r, Cohen's d)
- Report exact p-values wherever possible alongside the summary statistics and 95% confidence intervals. These should be reported for all key questions and not only when the p-value is less than 0.05.

Please outline where this information can be found within the submission (e.g., sections or figure legends), or explain why this information doesn't apply to your submission:

Statistical analysis methods are described in the Methods section; the normality of data was assessed through the Shapiro-Wilk normality test. Statistical significance ($p < 0.05$) was determined with non-parametric Wilcoxon matched-pairs signed rank test or Kolmogorov-Smirnov test when comparing two groups, and two-way ANOVA test for comparing multiple groups in conjunction with post hoc Tukey's HSD test for pairwise comparison (Prism, GraphPad software LLC). The specific test used for each comparison is reported in the Results section. We reported exact p-values. Summary data are presented as the mean ± SEM.

All of the experimental data values used for statistical analysis are presented in the Source data file.

(For large datasets, or papers with a very large number of statistical tests, you may upload a single table file with tests, Ns, etc., with reference to sections in the manuscript.)

Group allocation

- Indicate how samples were allocated into experimental groups (in the case of clinical studies, please specify allocation to treatment method); if randomization was used, please also state if restricted randomization was applied
- Indicate if masking was used during group allocation, data collection and/or data analysis

Please outline where this information can be found within the submission (e.g., sections or figure legends), or explain why this information doesn't apply to your submission:

eLife Sciences Publications, Ltd is a limited liability non-profit non-stock corporation incorporated in the State of Delaware, USA, with company number 5030732, and is registered in the UK with company number FC030576 and branch number BR015634 at the address Westbrook Centre, Milton Road, Cambridge CB4 1YG.

Group allocation was described in the Methods section; the Cre-dependent transgenic mice (VgluT2-tdTomato-ChR2-EFYP) were allocated to the optogenetic/pharmacological experimental group. VgluT2-tdTomato, non-ChR2-expressing transgenic mice were used for the control experiments.

No randomization/masking was applied, because the light effect in ChR2-expressing animals was very significant and entirely absent in non-expressing animals.

Additional data files ("source data")

- We encourage you to upload relevant additional data files, such as numerical data that are represented as a graph in a figure, or as a summary table
- Where provided, these should be in the most useful format, and they can be uploaded as "Source data" files linked to a main figure or table
- Include model definition files including the full list of parameters used
- Include code used for data analysis (e.g., R, MatLab)
- Avoid stating that data files are "available upon request"

Please indicate the figures or tables for which source data files have been provided:

We have uploaded numerical data Excel files as "Source data" file for the graphs in Figures 1-5, 7, 8, and Figure 1-figure supplement 1 and Figure 4-figure supplement 1.

We have also provided a source code file as a supplemental file.

eLife Sciences Publications, Ltd is a limited liability non-profit non-stock corporation incorporated in the State of Delaware, USA, with company number 5030732, and is registered in the UK with company number FC030576 and branch number BR015634 at the address Westbrook Centre, Milton Road, Cambridge CB4 1YG.
